# Acromegaly treatment and bone: a bidirectional relationship

**DOI:** 10.1007/s11102-025-01573-6

**Published:** 2025-11-08

**Authors:** Sabrina Chiloiro, Chiara Palumbo, Antonella Giampietro, Laura De Marinis, Antonio Bianchi, Andrea Giustina, Alfredo Pontecorvi

**Affiliations:** 1 School of Medicine and Surgery, Università Cattolica del Sacro Cuore, Rome, Italy; 2 Department of Medicine, Endocrinology and Diabetology, Fondazione Policlinico Universitario A. Gemelli IRCCS, Largo A.Gemelli, Rome, Italy; 3 Institute of Endocrine and Metabolic Sciences, Università Vita-Salute San Raffaele, IRCCS Ospedale San Raffaele, Milan, Italy

**Keywords:** Acromegaly, Bone, Vertebral fractures, GH, IGF-I, First generations somatostatin receptor ligands (octreotide, lanreotide), Second generation somatostatin receptor ligands (pasireotide), GH receptor antagonist (pegvisomant)

## Abstract

Acromegaly is a rare disease caused by the elevated and autonomous secretion of growth hormone (GH) from a pituitary somatotroph tumor or neuroendocrine tumors, and the subsequent hypersecretion of insulin-like growth factor I (IGF-I) in peripheral tissues. Excess GH and IGF-I cause several chronic and systemic complications that impact mortality, morbidity, and quality of life in patients with acromegaly. Excess GH and IGF-I play a crucial role in bone remodeling by increasing osteoclastogenesis and impairing osteoblastogenesis. Several studies have demonstrated an increased prevalence and incidence of fragility vertebral fractures (VFs) in patients with acromegaly. Long-term exposure to high levels of GH and IGF-I is recognized as a risk factor for fragility fractures in patients with acromegaly. Recent studies have shown that first- and second-generation somatostatin receptor ligands (SRLs) can reduce the incidence of vertebral fractures (i-VFs). However, a direct effect of these molecules on bone metabolism has not yet been reported. Aims: This review summarizes the results of studies investigating the frequency of i-VFs according to different GH/IGF-I-lowering drugs and the potential effects of these treatments on bone metabolism, as well as preclinical data on potential molecular pathways that interact between GH/IGF-I-lowering drugs and bone metabolism.

## Introduction

Acromegaly is a rare neuroendocrine disease, due to the elevated and autonomous secretion of the growth hormone (GH) from pituitary adenoma/neuroendocrine tumor. GH excess is able to increase circulating levels of insulin-like growth factor I (IGF-I), which is secreted by liver [1].

Acromegaly is a chronic disease with several comorbidities, including somatic alterations and cardiovascular disorders (systemic arterial hypertension, myocardial hypertrophy, mitral and aortic valvopathy, and arrhythmias). It also causes dyslipidemia (hypertriglyceridemia and reduced high-density lipoprotein cholesterol levels) and disorders of glucose metabolism (hyperglycemia, impaired glucose tolerance, impaired fasting glucose, diabetes mellitus), hypopituitarism, an increased occurrence of second neoplasms, sleep apnea syndrome and musculoskeletal disorders [2–6]. Increased bone resorption and the impaired bone formation cause the deterioration of bone microarchitecture and the increased risk of fragility fractures in patients with acromegaly [7]. Incident vertebral fractures (i-VFs) are reported in 20-60% of patients with acromegaly [8].

### Bone health in patients with acromegaly

GH and IGF-I play crucial roles in bone remodeling. GH both directly and indirectly through IGF-I, stimulates osteoblast proliferation and differentiation [9–11]. High levels of GH and IGF-I result in an increased osteoclastogenesis and bone resorption, also through the enhanced synthesis of receptor activator of nuclear factor-κB ligand (RANK-L) [12–16]. Furthermore, the same cytokines, such as the interleukin-6 (IL-6), RANK-L and the tumor necrosis factor (TNF) appear to be involved in bone resorption [10, 17, 18]. Other studies have demonstrated that in patients with acromegaly, increased bone resorption coexists with the increased bone neoformation, with a positive correlation between levels of circulating IGF-I and levels of bone formation markers (such as osteocalcin) and bone resorption markers (such as β cross laps) [19]. However, markers of bone formation appear to be less elevated than markers of bone resorption [20]. Therefore, in patients with acromegaly the increased osteoclastogenesis and the impaired osteoblastogenesis play crucial roles in developing abnormalities in bone microstructure, particularly in trabecular bone [9, 21], resulting in a loss of bone’s strength and an increased risk of fragility fractures [16].

Several studies have examined the prevalence and incidence of fragility fractures in patients with acromegaly. A cross-sectional study on 36 postmenopausal women showed that VFs occurred in 52.8% of cases, primarily among patients with active acromegaly (80% versus 33.3%) [22]. Another cross-sectional study on 40 male patients with acromegaly found that the prevalence of VFs was 57.5% [23]. In a case-control study by Wassenaar et al., on 89 patients, the prevalence of VFs was 59%, resulting quite higher in males (64%) then in females (54%) [24]. In other studies, the prevalence of VFs was lower, ranging from 10.6% to 39% of study cohorts [25–27]. A meta-analysis conducted by Mazziotti et al. on a total of 473 patients, reported a frequency of radiological VFs in acromegalic patients of about 38%, with a three times higher risk for the occurrence of VFs in patients with uncontrolled disease than in those who have achieved control of GH and/or IGF-I levels [20]. In fact, the impaired osteoblastogenesis persists also in patients with well-controlled acromegaly disease [12–16], as proven by the persistence of high serum concentrations of bone turnover markers, albeit at lower concentrations than in patients with biochemical active disease [20].

Several studies have investigated the risk factors associated with the occurrence of VFs in patients with acromegaly. These studies identified as main risk factors the longer duration of biochemically active disease, the longer diagnostic delay, the presence of preexisting VFs, the untreated hypogonadism, the diabetes mellitus and the male gender, as reported in table 1 [8, 20, 28–32]. More recently, vitamin D deficiency, which is frequently observed in patients with acromegaly [33], was associated with a high frequency of i-VFs. [34]. Indeed, bioavailability of 25-hydroxy (OH)-vitamin D is reduced in patients with acromegaly. Excess GH and IGF-I modulate the secretion of vitamin D with genomic and not-genomic mechanisms: GH and IGF-I directly affect the liver microsomal enzyme system, regulate intestinal calcium and phosphate absorption, stimulate renal calcium reabsorption and 1,25(OH)2-D vitamin production and inhibit the parathyroid hormone (PTH) secretion [35]. IGF-I, phosphate, PTH and fibroblast growth factor 23 (FGF-23) stimulate renal production of 1,25OH2-D vitamin, which increases calcium and phosphate availability in the body and suppresses PTH secretion [35]. The 25-OH vitamin D levels are reduced in patients with biochemical active acromegaly, due to increased levels of circulating vitamin D binding protein (VDBP) [33, 36–40]. Additionally, some therapies, such as somatostatin receptor ligands (SRLs), decrease the intestinal lipid absorption and contribute to hypovitaminosis D in these patients [41, 42]. The vitamin D supplementation reduces the frequency of VF in patients with acromegaly [43].

### Effects of medical therapies on bone metabolism: data from human real-life studies

Preliminary data on the effects of acromegaly treatments on bone metabolism are available. Considering that the therapeutic options for the treatment of acromegaly was significantly enlarged in the last 20 years [44–48], the potential effects of GH and IGF-I lowering drugs on bone health were investigated in patients with acromegaly. Although the data are limited, some studies have focused on assessing the frequency of i-VFs during treatment with first- generations and second- generation SRLs (respectively fg-SRLs and sg-SRLs), with GH receptor antagonist. This review aims to provide a comprehensive overview of the available literature, according to different medical treatments, and to review the mechanisms of action and potential effects on bone metabolism, as well as laboratory assessments, that can help clarify the impact of these therapies on bone metabolism.

### First generation somatostatin receptor ligands (fg-SRLs) and bone

Native somatostatin regulates hormones synthesis and secretion from the anterior pituitary gland, the pancreas and cells of the amine precursor uptake and decarboxylation (APUD) system. The peripheral effects of somatostatin are mediated by five different somatostatin receptor (SSTR) subtypes: the SSTR1, SSTR2, SSTR3, SSTR4 and SSTR5 [49]. Binding with somatostatin receptors, activates of the cytoplasmic intracellular signal transduction, which regulates apoptosis, cell proliferation and hormone secretion. The same mechanism is mediated by the synthetic analogues, such as fg-SRLs and sg-SRLs [49]. Binding of native or synthetic somatostatin to its receptors causes the inhibition of calcium (Ca^2+^) channels and the activation of potassium (K^+^) channels. This leads to a decrease in intracellular Ca^2+^ concentration (mainly via SSTR1, SSTR2 and SSTR5), inhibition of adenylyl cyclase, and a subsequent decrease in cyclic adenosine monophosphate (cAMP) and consequently in a reduction of hormone secretion [50, 51]. SRLs reduce cell proliferation by inhibiting the phosphatidylinositol 3-kinase (PI3K)/protein kinase B (PKB or AKT) signaling pathway through SSTR2 or by modulating the mitogen-activated protein kinase (MAPK) pathway mainly via SSTR1, SSTR2, and SSTR5 [51–53]. Furthermore, SRLs appear to increase apoptosis by binding to SSTR2 and SSTR3 and acting on the nuclear factor kappa-light-chain enhancer of activated B cells (NF-kB)- c-Jun N-terminal kinase (JNK) -caspase pathway [51, 52].

In GH-secreting pituitary adenomas, SSTR2 is detected in 95% of cases, SSTR5 in 85% of cases, SSTR1 and SSTR3 are identified in approximately 40% of cases, while SSTR4 is rarely detected [54, 55]. Fg-SRLs (octreotide and lanreotide) bind SSTR2 with high affinity and the SSTR3 and SSTR5 with weak to moderate affinity [51, 56–58]. Fg-SRLs are indicated for the treatment of patients who have undergone non-curative surgery or as a primary treatment (an alternative to surgery) in limited clinical scenarios, such as in patients with contraindications to general anesthesia or major surgery and in patients who decline surgery. Although several studies over the last 30 years have largely proved the efficacy of fg-SRLs in achieving the biochemical control of acromegaly in 45-55% of patients [59], few clinical and laboratory data are available on the effects of fg-SRLs on human bone metabolism [60]. Table 2 summarizes the sites of SSTR expression in animal experiments and in humans.

Few studies in literature have investigated the frequency of VFs in patients with acromegaly undergoing treatment with fg-SRLs. In a retrospective and longitudinal multicenter study, conducted on 42 patients with acromegaly, the incidence of VFs during treatment with fg-SRLs was around 44% in patients with active acromegaly and around 26% in patients with controlled disease [60]. However, clinical and laboratory data on the potential direct effects of fg-SRLs on bone metabolism are limited to preclinical studies. Vitali et al. recently investigated the effects of octreotide on the proliferation of murine osteoblastic and pre-osteblastic cells [61], demonstrating that octreotide reduced cell proliferation in pre-osteoblastic and osteoblastic cells and increased apoptosis in pre-osteoblastic cells. After silencing the *SSTR5* and *SSTR2* genes in pre-osteoblastic cells, the authors proved that the anti-proliferative effect of octreotide was abrogated in *SSTR2-* and *SSTR5-*silenced cells, and that the pro-apoptotic effect of octreotide was abrogated only in *SSTR2-*silenced pre-osteoblastic cells [61]. Therefore, the results of this study suggested that the anti-proliferative effect of octreotide was mediated through SSTR2 and SSTR5; however, the pro-apoptotic effect was mediated only through SSTR2 [61]. Octreotide did not impact on the expression of the genes *Runt-related transcription factor 2 (Runx2), Alkaline phosphatase (Alp), secreted phosphoprotein 1 (Spp1), Bone Gamma-Carboxyglutamate Protein (Bglap), Rankl, osteoprotegerin (Opg), Sclerostin (Sost)* genes, but it reduced the expression of the vascular endothelial growth factor (*VEGF*) gene [61]. However, data on humans instead are lacking as reported in the genomic database, the SSTR gene has not yet been identified in human bone cells [62].

### Pasireotide Long Acting Release (Pasireotide LAR) and bone

Pasireotide LAR is a multireceptor-targeted SRLs, able to bind to four SSTRs, mainly subtypes 1,2,3 and 5 of the SSTR, with the highest affinity for the SSTR5 [51]. Prospective, randomized trials and several post-marketing real-life studies confirmed data on the efficacy of pasireotide LAR in managing acromegaly patients who do not respond to fg-SRLs [63, 64]. In a recent metanalysis, the rate of IGF-I control was 56% and the rate of tumor shrinkage was of 41% [65]. Pasireotide has been shown to have a greater biochemical effect on tumors that express a higher level of SSTR5, consistent with its high binding affinity for this SSTR subtype. In a study conducted by Iacovazzo et al., no patients with SSTR5-negative tumors were responsive to pasireotide LAR [66]. However, although preliminary studies showed that pasireotide acts via SSTR5 [51, 66], recent evidence showed that pasireotide LAR acts also via SSTR2 [67]. In a recent study conducted by Muhammed et al., an inverse correlation was proved between the immunoreactive score (IRS) and the reduction of IGF-I levels, in a cohort of 14 patients treated with pasireotide LAR and with available data on the pituitary tumor SSTR2A expression. This suggests that the high SSTR2A expression predicts a good response to pasireotide LAR treatment, and that the response to pasireotide is also driven by SSTR2, in patients who have not been treated with fg-SRLs [67]. Pasireotide LAR modulates SSTR activity differently from octreotide, maintaining SSTR expression on the cell membrane [68, 69].

Studies investigating the potential effects of pasireotide LAR on bone metabolism are limited. A longitudinal, retrospective international study examined the frequency of incidental VFs in a group of 24 patients with acromegaly who were treated with pasireotide LAR [70]. This study proved that 25% of patients with active acromegaly experienced incidental VFs, compared to 10% of patients with biochemical controlled disease. Furthermore, this study demonstrated also that the only risk factors for the occurrence of new VFs was the presence of pre-existing VFs, rather than the IGF-I levels, despite in the study population, eight patients were affected by active disease at last-evaluation [70]. Recently, the association between GH receptor (GHR) polymorphisms and the occurrence of incidental VFs was investigated in patients treated with Pasireotide LAR [71].

Different GHR isoforms have been reported with different sensibility to the GH stimulation [19]. In fact, the exon 3 delated isoform is more sensitive to GH stimulation [19]. Conversely, acromegaly patients with the exon 3 deleted GHR (d3-GHR) isoform have a higher prevalence of VFs, regardless of disease activity. This is possibly due to the increased receptor affinity and sensitivity to GH stimulation [19, 72, 73]. Furthermore, GH receptor isoforms influence the efficacy of medical therapies. Patients with the d3-GHR isoform had a poor response to pasireotide LAR [19, 74] and an increased risk of VFs [71]. In fact, a recent study found that VFs mainly occurred in patients treated with pasireotide LAR who carried the d3-GHR isoform [71].

### Pegvisomant and bone

Pegvisomant, a GH receptor antagonist, was derived from modifications to the human GH molecule [75–78]. Pegvisomant differs from human GH due to a glycine substitution in the third alpha-helix (the receptor-binding site), which prevents its interaction with one of the GH receptor subunits [79, 80]. Additional substitutions were added to enhance the affinity of pegvisomant for the receptor in the other binding site [79, 80]. Finally, four to six polyethylene glycol (PEG) moieties were added to increase its half-life [75]. Thus, pegvisomant antagonizes endogenous GH, blocks the receptor, and reduces IGF-I production, thereby improving clinical outcomes [48].

The efficacy of pegvisomant in controlling acromegaly is well recognized and reported in several studies. In a recent analysis of the ACRO-STUDY registry conducted on 2.221 patients, over 79% of patients reached the biochemical control [81]. The effects of pegvisomant treatment on human bone health have been also investigated, but clinical and laboratory data are limited. During treatment with pegvisomant monotherapy or in combination with fg-SRLs, the frequency of i-VFs ranged from 25% to 78% in patients with active disease and from 16.7% to 29% in patients with well-controlled disease [70, 82].

A longitudinal, retrospective, international study investigated the frequency of i-VFs in a cohort of 31 patients treated with pegvisomant in association with fg-SRLs with a minimum follow-up period of 15 months [70]. Among patients with active acromegaly at last follow-up 77.8% developed i-VFs. In contrast, among patients with biochemical controlled disease, i-VFs occurred in 29.4% of cases. This study also proved that in patients treated with pegvisomant who had pre-existing or prevalent VFs, active disease, or high levels of IGF-I were at an increased risk of new or i-VFs [70]. These clinical data were recently confirmed in a retrospective cross-sectional study, showing that patients with acromegaly experienced a significant reduction in trabecular bone density, after nine years of pegvisomant treatment [83].

A study by Vitali et al. suggested that pegvisomant alone cannot directly modulate the proliferation, differentiation, or apoptosis of murine preosteblastic or human osteoblastic cells. In the same study, the authors proved that pegvisomant appear to modulate osteoblastic cell proliferation and differentiation after GH stimulation. Activation of the GH signaling pathway enhances the pegvisomant action on pre-osteblastic and osteoblastic cells. This study also demonstrated a GH-induced reduction in cell apoptosis, and showed that the addition of pegvisomant can reverse this process [84]. Furthermore, GH and pegvisomant did not affect local IGF-I secretion in murine pre-osteoblastic cells [84]. This phenomenon could explain why the bone remodeling process persists also in patients with well-controlled acromegaly [84, 85]. The study also evaluated the effect of pegvisomant on the expression of *Alp*, *Runx2*, *Opg* and *Spp1* genes. There was no difference in gene expression between cells exposed to pegvisomant and those not exposed. Additionally, pegvisomant alone or in combination with GH did not affect the expression of *genes IGF-I, Insulin-like growth factor binding protein 2 (IGF-BP2) and Insulin-like growth factor binding protein 4 (IGF-BP4)* [84].

Moreover, as already mentioned in previous paragraph, GH receptor isoforms influence the outcome of medical therapies. Patients carrying full length GHR isoform have an increased fracture risk when treated with Pegvisomant in combination with fg-SRLs and a reduced risk of i-VFs when treated with Pasireotide LAR, reflecting the different affinity for GH of the full length and d3-GHR isoforms [71, 86].

### Combination therapy pegvisomant plus pasireotide LAR and bone

Data on the potential effects on bone health of the combination therapy pegvisomant plus pasireotide LAR are limited to a small case series, derived from the real-world retrospective and longitudinal experience. This combination therapy is limited to patients not responsive to conventional therapeutic regimens. In a recent real-world case series of six patients on combination pasireotide LAR plus pegvisomant, no patients experienced i-VFs, despite the expected aggressive and multi-drugs acromegaly history [82].

### Clinical perspective

**Fig. 1:**
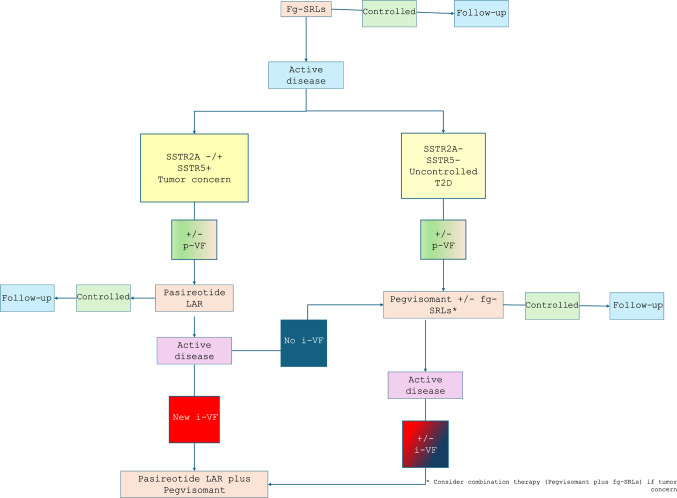
Flow chart for the choice of GH/IGF-I medical therapies in patients with acromegaly, according to molecular tumor profile and vertebral fractures occurrence. Abbreviations: fg-SRLs: first-generation somatostatin receptor ligands, SSTR: somatostatin receptor; T2D: type 2 diabetes; i-VF: incidental vertebral fracture

Evidence from these retrospective, real-life studies may be of clinical interest and may guide personalized medical therapy for patients with acromegaly, because medical therapy with SRLs and pegvisomant were proved to correct high bone turnover, partially recovery the bone quality, although microstructural abnormalities persisted and the risk of i-VFs remained increased [87]. These studies suggest that the choice of second-line drugs can be guided from molecular profile of the tumors, and patient clinical assessment, such as the presence of dyslipidemia, hyperglycemia, and skeletal fragility. Although available studies have not demonstrated a direct effect of GH/IGF-I-lowering therapy on bone metabolism, the treatment with fg-SRLs, pegvisomant and pasireotide LAR seem to have a neutral effect in patients with controlled disease. However, patients with active disease seem to benefit from the treatment with pasireotide LAR alone or in combination with pegvisomant, if clinically indicated. Therefore, as we summarized in Fig. 1, in patients with biochemical active disease during treatment with fg-SRLs, the occurrence of incidental VFs should orient the choice of second- and third-line medical therapies, also according to SSTR tumor expression. Patients not responsive to fg-SRLs, with somatotroph tumors with SSTR2A and/or SSTR5 expression may response to treatment with Pasireotide LAR [88]. Instead, patients with somatotroph tumors without SSTR2A and SSTR5 expression and/or with uncontrolled type 2 diabetes should be treated with pegvisomant in monotherapy or in combination with fg-SRLs. Therefore, in patients with active disease during pasireotide LAR therapy, the occurrence of i-VFs may suggest treating the patient with the combination therapy pasireotide LAR plus pegvisomant; instead, the absence of i-VFs may suggest treating the patient with the combination therapy fg-SRLs plus pegvisomant, according to previous studies [82]. Patients not-responsive to the pegvisomant alone or in combination with fg-SRLs and with absence expression of SSTR2A and SSTR5 may be treated with the combination pasireotide LAR plus pegvisomant independently of the occurrence of i-VFs, according to the protective role of pasireotide LAR on bone in patients with biochemical active disease.

These patients with difficult-to-treat acromegaly and with skeletal fragility should be referred to a team of bone expert in a pituitary tumor center of excellence (PTCOE), to assess the risk of skeletal fragility, and schedule the most appropriate diagnostic workflow, follow-up and treatment options. This is according to the limited evidence on bone active drugs and on GH/IGF-I lowering treatments in acromegaly [89, 90] .

## Conclusion

Skeletal fragility is a significant complication of acromegaly, that requires a prompt screening and treatment. In the era of the personalized therapy, the effects of GH/IGF-I lowering therapies on bone health may also be considered when choosing the most effective treatment for the management of a chronic and systemic disease, such as acromegaly. Additional basic, translational, and randomized controlled trials (RCTs) are needed to validate current evidence and understand the molecular mechanisms involved in the drug-mediated effects on bone health in patients with acromegaly.

## Data Availability

No datasets were generated or analysed during the current study.
